# Effect of rh-BMP-2 in the Initiation of Neovascularization in Human Gingival Tissue: A Split-Mouth Clinical Study

**DOI:** 10.3390/life13122298

**Published:** 2023-12-04

**Authors:** Mansour Chantiri, Samir Nammour, Sami El Toum, Toni Zeinoun

**Affiliations:** 1 Department of Periodontology, Faculty of Dental Medicine, Lebanese University, Beirut 27798, Lebanon; mansour-chantiri@ul.edu.lb; 2 Department of Dental Sciences, Faculty of Medicine, University of Liege, 4000 Liege, Belgium; 3 Department of Oral Medicine and Maxillofacial Radiology, Faculty of Dental Medicine, Lebanese University, Beirut 27798, Lebanon; samitoum@ul.edu.lb; 4 Department of Oral and Maxillofacial Surgery, Faculty of Dental Medicine, Lebanese University, Beirut 27798, Lebanon; toni.zeinoun@ul.edu.lb

**Keywords:** recombinant human bone morphogenic protein 2, vascular endothelial growth factor, gingival tissue, guided bone regeneration, neovascularization

## Abstract

The aim of this study is to evaluate the effect on the initiation of new blood vessel formation of rh-BMP-2 administration in the human gingival tissue during bone regeneration surgery. Material and Methods: The randomized controlled clinical trial included twenty patients with bilateral partial edentulous of the mandibular premolar and molar region. Each patient received one implants on each side. Only one side received a 0.25 µg injection of rhBMP-2 into the gingival flap and grafted material during guided bone regeneration (GBR) for dental implantation. And the other side received GBR without injection. Three samples were collected from each patient as follows: one from the anterior area of the mandible (control group #1) collected at the time of all implant surgeries, and the two other samples during the placement of healing abutments at 4 months of follow-up, from treated side with rh-BMP-2 (test group) and untreated ones (control group #2). A total of 60 gingival samples were collected. Samples were stained with hematoxylin-eosin, and immunohistochemistry was performed with a vascular endothelial growth factor marker. The number of new vessels in each sample was counted. Result: Statistical analyses showed a significantly higher number of new vessels in the gingival tissue of the test group. Conclusions: Rh-BMP-2 injections into the gingival flap significantly improved new blood vessel formation.

## 1. Introduction

Bone morphogenic proteins, known as BMPs, are a group of about 20 proteins, which belong to the transforming growth factor-β family. These glycoproteins stimulate mesenchymal cells and help their differentiation into osteoblasts. They also regulate many cellular activities such as migration, differentiation, and proliferation (fibroblasts, endothelial, and osteoblasts). BMPs are involved in many intracellular pathways intersecting with oncogenic pathway proteins involved in the development of many tumors, especially those invading and colonizing bone tissues [1].

Since the discovery of BMPs as bone-inductive proteins by Urist [1], many investigators stated that BMPs induce stem and mesenchymal cell differentiation into osteogenic cells, which produce bones. According to current evidence in molecular biology, BMPs are morphogenetic proteins or molecules that induce the initiation of morphogenetic area formation [2]. BMPs play a crucial role in the cascade of bone formation during the development and bone fracture repair [2]. Furthermore, BMP-2 is frequently used in dentistry for a variety of procedures, such as periodontal regeneration, sinus lift augmentation, dental implant surfaces, and alveolar bone regeneration. Additionally, growth factor delivery times are extended by BMP-encoding gene therapy vectors, whereas they decrease the functional dose needed to treat a local deficiency [3].

Currently, at least 15 distinct BMPs have been identified as components of the TGF-β superfamily. All recombinant BMP types have already been synthesized, such as recombinant human BMPs (rh-BMPs). The sequences of BMPs and TGF-β are similar and BMPs are classified as regulatory genes [4,5].

BMPs are involved in the extracellular matrix during repair and regeneration. They also stimulate angiogenesis by extracellular matrix interactions [6,7]. The term angiogenesis was first used in 1971 by Folkman [8] and is defined as the process of new blood vessel formation from pre-existing vessels. It is an essential process for development and growth, as well as in bone formation [9].

Many angiogenic activators are reported, such as the vascular endothelial growth factor (VEGF), which is considered as a potent angiogenic agent in both tumoral and normal tissues [6,7]. Tumors can grow by forming new blood vessels from the existing vascular system, and angiogenesis is closely related to tumors and various other diseases [8,9,10].

VEGF is a highly potent angiogenic agent that increases vessel permeability and enhances endothelial cell growth, proliferation, migration, and differentiation [11,12,13]. The VEGF family is expressed in tumoral tissues and is crucial in neovascularization under the effect of other growth factors and specific cytokines [14].

VEGF-A has at least six molecular isoforms. It is a heparin-binding glycoprotein and a potent and selective mitogen for vascular endothelial cells. It stimulates the entire signaling cascade required for angiogenesis [15]. Radovic et al. [16] highlighted the importance of VEGF in gingival healing in patients with type 2 diabetes.

BMP-2 is a member of the VEGF family and induces angiogenesis by stimulating endothelial cells, enhancing tube formation, inducing phosphorylation, and increasing Id1 expression, which leads to endothelial cell activation [17]. Furthermore, BMPs enhance angiogenesis through VEGF-A release by osteoblasts [18].

Angiogenesis is observed in inflammatory reactions, wound healing, and immune reactions. During tissue healing, new blood vessel growth is essential for gingival healing [19]. At the gingival level, several variables can be used to evaluate the impact of recombinant human bone morphogenetic protein proliferation, such as the percentage index of the VEGF isoforms or Ki-67 in the gingival tissue [20].

The aim of this study is to evaluate the effect of a rhBMP-2 injection in the gingival tissue on new blood vessel formation. The null hypothesis is that gingival injection of rh-BMP-2 would produce no significant change in new blood vessel formation.

## 2. Materials and Methods

The study protocol was approved by the ethical committee of the Lebanese university under the number CUER 27-2020. The randomized control trial was registered (LU-DP-5.2.20).

### 2.1. Study Design

**Figure 1:**
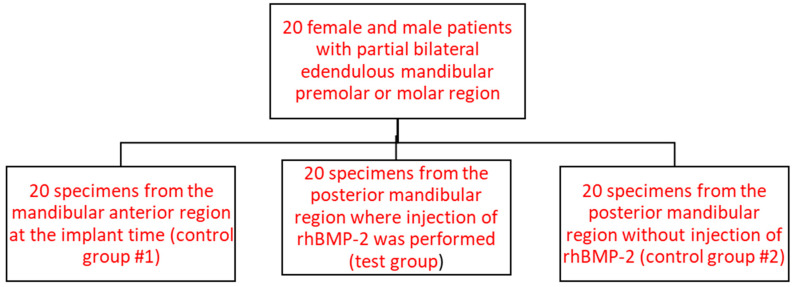
Schema of the study design.

This study was a clinical and histological investigation in which 20 patients were consecutively included according to inclusion and exclusion criteria, at Lebanese University. This randomized control trial included three groups. A total of 60 gingival samples were collected (three per patient, one for each group): 20 samples from the normal gingivae of the anterior mandible region (control group #1), 20 samples treated with 0.25 µg of rhBMP-2 during guided bone regeneration (GBR) and dental implantation in the posterior mandible region (test group), and 20 samples without rh-BMP-2 administration during GBR on the opposite posterior mandible region of the same patient (control group #2) (Figure 1). The samples for control group #1 were collected from the mandibular anterior site at the time of implant placements and samples for the test group and control group #2 were collected from the posterior mandibular posterior region, during the abutment placement after 4 months of follow-up.

Overall, 20 healthy patients with an average age of 28 years old (range: 20–60 years old) were included, irrespective of gender. All patients were selected according to inclusion criteria and signed a written informed consent. If a patient chose to withdraw from the study before implant placement, another one replaced him. A total of 29 patients were involved in this study, but only 20 met all the study’s inclusion criteria. All patients enrolled in the study were asked to participate in the investigation in consecutive order, if they fulfilled the criteria stated in this protocol.

### 2.2. Selection Criteria

Patients were selected with respect to the following criteria:

Inclusion criteria:
  - Males or females older than 18 years old.
  - Available for all study visits over 4 months.
  - Ability to provide written informed consent.
  - Needing bone augmentation in the posterior areas on both mandible sides during implant placement.
  - Non-smokers or light smokers (less than five cigarettes per day).
  - Patient with split-mouth edentulous mandibular posterior ridge with a bucco-lingual bone width of 6 mm or less, calibrated with cone-beam computed tomography (CBCT).
Exclusion criteria:
  - Patients at risk of infection: presence of one or more known infectious diseases (human immunodeficiency virus [HIV], hepatitis, infectious mononucleosis).
  - Known clinically important systemic disease.
  - Non-controlled diabetes.
  - Known risk of endocarditis.
  - Use of anti-thrombotic treatment (heparin, anti-vitamin K).
  - Severe difficulties in understanding written and spoken Arabic/English/French.
  - Chronic disorders requiring chronic or intermittent use of antibiotics.
  - Known hypersensitivity to BMPs.
  - Participation in another interventional study.
  - Known contraindications to both amoxicillin and clindamycin and to dental local anesthetics.
  - Pregnancy or lactation or intent to become pregnant.

### 2.3. Randomized Group Selection

In this randomized controlled trial, a split-mouth study was performed on patients who had lost at least one premolar or molar mandibular teeth from each side. The missing teeth were treated by implant placement with guided bone graft on the buccal or lingual side and injection or not of rh-BMP-2. It was decided randomly and before the start of this split-mouth study that the right side of the posterior mandible would be treated with rh-BMP-2 for all patients (test group).

The gingival area in the test group was treated with 0.1 mg of the Cowell Medi product with a concentration of 0.5 µg/mL of rh-BMP-2 during dental implantation and bone grafting in the posterior mandible area. The two other groups did not receive any rh-BMP-2 administration.

### 2.4. Implant Placement

With a 15c blade, an intra-sulcular incision was performed from the first existing tooth mesially to the edentulous ridge and continued posteriorly with a mid-crestal incision distally to the second molar position. A full-thickness mucoperiosteal flap elevation was performed. The drilling sequence for a 4.0 INNO implant with a length of 8 mm or 10 mm was performed with a series of drills of variable diameters, as recommended by the implant company. The Inno Implant (4.0) was placed with an implant holder using the handpiece with a torque of 30 N/cm.

After placing an implant of 4.0 mm diameter in a ridge of 6 mm or less, a bone dehiscence occurred. GBR procedure was recommended to cover the total exposed part of the implant on the buccal or lingual surface.

### 2.5. Guided Bone Regeneration Procedure

The GBR procedure involved a bovine bone graft (Dia Bone, Cowell Medi Co., Ltd., Seoul, Republic of Korea) with a cytoplast membrane (Dia membrane Cowell Medi Co., Ltd., Seoul, Republic of Korea).

The bovine bone grafting material was applied directly to the implant surface and covered with a membrane. The flap was sutured over the membrane with separated horizontal sutures.

On each right mandibular posterior side, a BMP injection (Cowell Medi R&D, Ref: BB1025, Seoul, Republic of Korea) in the gingiva and the grafting material was performed by using a syringe with a 21 G needle (Weigao Medical International Co, Ltd., Weihai, China) through the gingival tissue, with a fine needle infiltration to the bone graft.

Concerning control group 2, there was no injection of BMP on the left posterior side of the same patient. Both treatments were performed simultaneously in one session.

### 2.6. Second Implant Stage (4 Months Later)

The implant was localized by using a periodontal probe. The implant was uncovered using a traditional punch technique with a tissue puncher (Cowell Medi Co, Ltd., Seoul, Republic of Korea) with a 4 mm diameter. The gingiva specimen was then fixed in 10% buffered formalin and then sent to the laboratory for processing.

### 2.7. Immunohistochemistry

The samples were stained with hematoxylin-eosin, followed by immunohistochemical staining. VEGF expression was measured by counting the number of new blood vessels, as VEGF markers typically stain new blood vessels brown and existing blood vessels purple color, according to the National Center for Biotechnology Information at U.S. Information (N.C.B. I). Five slides were prepared and stained for each biopsy specimen. On each slide, the total number of blood vessels was counted, and the average count for the five slides was calculated. Subsequently, the mean value for each group was determined, as presented in Table 2.

#### 2.7.1. VEGF Antibody Staining Protocol for Immunohistochemistry

The primary antibody used in this study was the VEGF antibody. Its clone is VG1, rabbit “anti-Human” supplied by Chemi-Con Registered office: 40-42 Hatton Garden, London, England, EC1N. -An 8EB-Catalog Number: MAB3734.

#### 2.7.2. rh-BMP-2

We used 1 mL of rh-BMP-2 (0.25π g/mL) soluble in physiological serum (Cowell Medi Co., Ltd., Republic of Korea under the reference: BB1025) for our injections.

### 2.8. Histological Procedures and Immunohistochemical Examination

Tissues were fixed in 10% buffered formalin and sent to the histological laboratory. The specimen was embedded with paraffin. Two or three serial sections of 4 μm thickness were prepared and placed on salinized slides. The sections were then deparaffinized and rehydrated through xylene and three descending grades of alcohol. Antigen retrieval was carried out in a pressure cooker in 10 mM citrate buffer (pH 6.0) for 2 to 5 min. The sections were incubated after covering them with 3% hydrogen peroxide for 15 min to block any endogenous peroxidase activity, and then 100 slides were incubated with primary anti-VEGF-A rabbit polyclonal antibody (Abcam Inc., Cambridge, MA, USA) for 4 h at room temperature using an optimal dilution of 6 μg/mL. After further incubation with the secondary antibody (45 min) and streptavidin peroxidase (30 min), visualization was performed using freshly prepared diaminobenzidine (DAB) chromogen for 10 min.

Finally, the slides were examined under a microscope to investigate the staining patterns and VEGF expression in each group. In each slide of all groups, five fields of each slide were visualized under 40× magnification and used to count and distinguish new and pre-existing vessels.

### 2.9. New Blood Vessel Count

Under microscopic examination, the color of the new blood vessels was brown, and the original vessels were purple. The count of blood vessels-stained brown per field (millimeter square) indicated the number of new blood vessels and the same procedure was performed with the purple-color blood vessels. For each biopsy specimen, five slides were performed and stained, and five field counts were performed for each slide. The mean of the five slides for each specimen was calculated, allowing us to calculate the global mean and the standard deviation for each group.

### 2.10. Statistical Analyses

Statistical analyses were carried out with Prism 9.5.1. 733 ^®^ software (GraphPad Software, Inc., San Diego, CA, USA). For the analysis, a *p*-value < 0.0001 was considered statistically significant. The confidence level was proposed to be 99% with *p* > 0.001, which was highly significant. For descriptive statistics, mean and standard deviation values of the blood vessel counts were calculated for the different groups: normal gingiva without any treatment or surgery (control group #1), gingiva without rhBMP-2 during GBR (control group #2), and gingiva with rhBMP-2 administration during GBR (test group). Moreover, the normal distribution of variables was assessed using normality tests: D’Agostino; Pearson, Anderson–Darling, Shapiro–Wilk, and Kolmogorov–Smirnov tests. In the case of non-normal distributions, Friedman tests for non-parametric and repetitive measurements coupled to the Dunn’s multiple comparisons test (ad hoc test) were used.

## 3. Results

The immunochemistry examination with VEGF markers-stained new blood vessels brown and the existing blood vessels purple. Enhanced brown staining indicated an increased number of new blood vessels, whereas an increase in purple staining assessed the presence of a normal vascularization network without the presence of a neovascularization.

**Figure 2:**
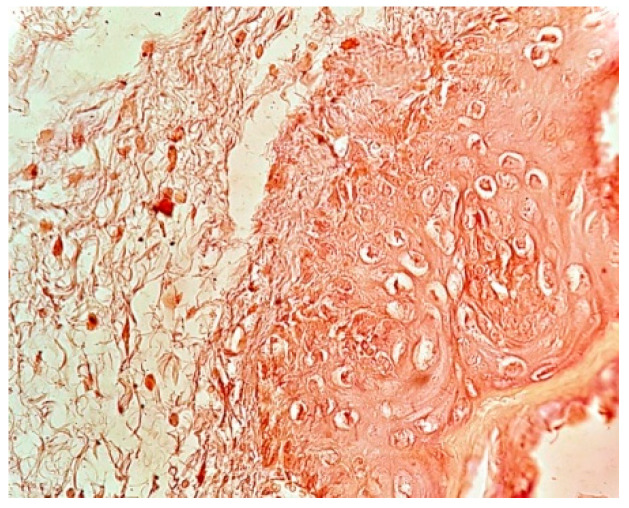
View of strong brown staining intensity in a gingival sample, with bone graft and rh-BMP-2 infiltration, stained with VEGF antibody. Magnification: 40×.

In the test group (Table 1), 80% of the samples (*n* = 16) displayed a strong brown staining intensity as shown in Figure 2 and 20% (*n* = 4) exhibited a lower intensity and were considered as moderate intensity, as illustrated in Table 2.

**Figure 3:**
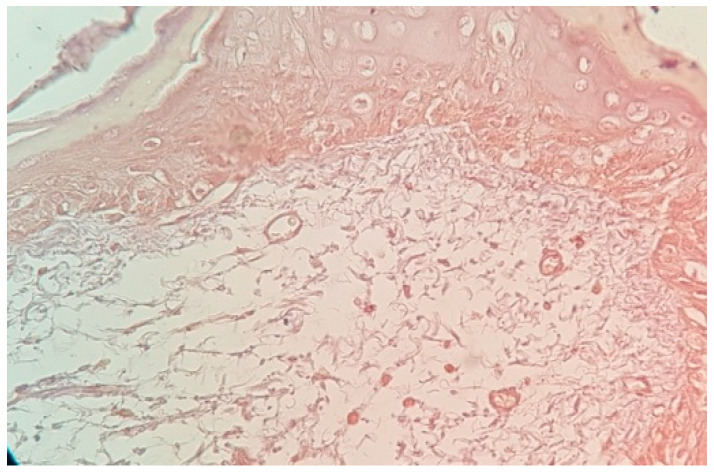
View of moderate brown staining intensity in a gingival sample, with bone graft (control group #2) without rh-BMP-2, stained with VEGF antibody. Magnification: 40×.

In control group #2 (without rh-BMP injection), 20% of the samples (*n* = 4) displayed moderate brown staining intensity (Figure 3), whereas 80% of samples (*n* = 16) exhibited weak brown staining intensity.

**Figure 4:**
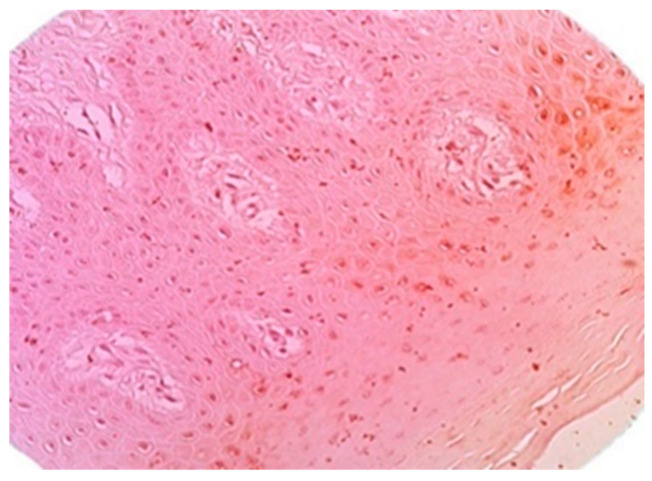
View of the purple staining in a normal gingival sample (control group #1), stained with VEGF antibody, indicating the presence of existing vessels. Magnification: 40×.

In control group #1, the intensity of purple was noted in all cases (Figure 4, Table 1).

The test group (rh-BMP-2-injected group) showed a strong brown staining intensity in 80% of samples, indicating a high number of new blood vessels, which improved bone graft vascularization more than in control group #2, grafted without rhBMP-2, or control group #1. A moderate brown color was observed in 20% of samples and indicated a lower number of new blood vessels, and 80% of the samples expressed a pale purple color. In control group #1, the absence of brown staining revealed the absence of new vessel formation.

The descriptive results of this study showed that gingival tissue vascularization and new blood vessel formation were significantly higher in the rh-BMP-2 group (test group) than in the control group. The total blood vessel counts per field (/mm^2^) was significantly different among the groups. The rh-BMP-2 group (test group) displayed a higher average percentage (86.71 ± 1.838%) of total blood vessels counted per field (/mm^2^) than the grafted group without rh-BMP-2 (control group #2), with an average of 78.07 ± 1.903%, and the normal gingiva group (control group #1), with an average of 51.14 ± 2.391% (Table 2).

**Figure 5:**
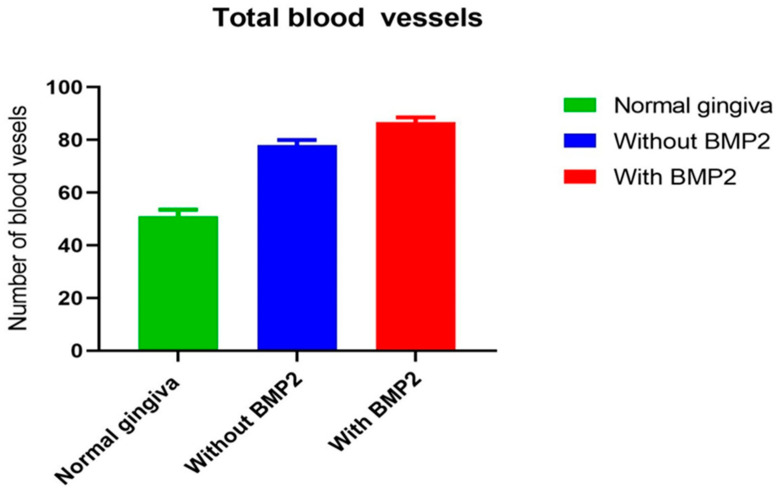
Mean total blood vessel count in the three groups. BMP-2: bone morphogenic protein 2.

Figure 5 illustrates that the test group had the highest mean number of vessels per field (/mm^2^). The results of this study showed that the rh-BMP-2 enhanced bone graft vascularization.

Moreover, the distribution of all vascularization data values in each group did not pass the normality tests (D’Agostino, Pearson, Anderson–Darling, Shapiro–Wilk, and Kolmogorov–Smirnov tests). Friedman tests for non-parametric and repetitive measurements coupled to Dunn’s multiple comparisons tests (ad hoc test) showed a high significant difference in the total blood vessel count among all groups (*p* > 0.0001) (Table 2).

**Figure 6:**
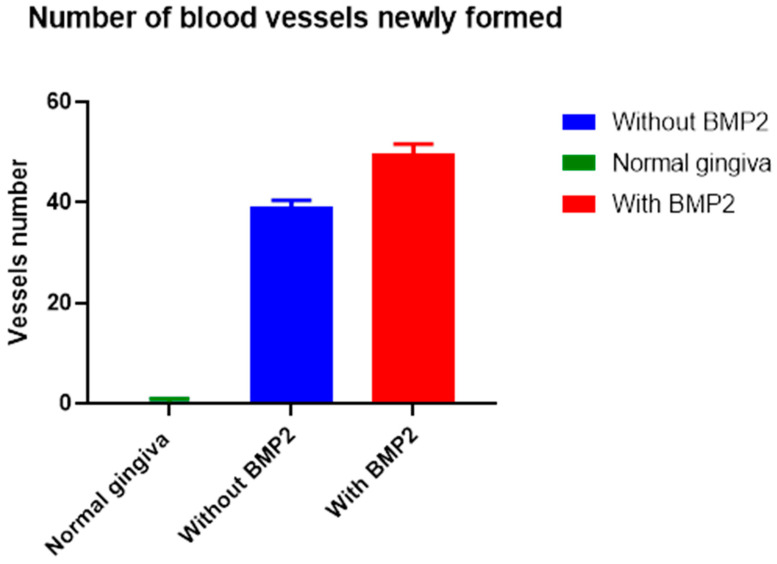
Average of new blood vessels in the three groups. BMP-2: bone morphogenic protein 2.

On the other hand, counting only new blood vessels showed that the rh-BMP-2 group (test group) exhibited a significantly higher new blood vessel count per field (/mm^2^) with a mean of 49.80 ± 1.809% than the grafted group without rh-BMP-2 (control group #2) with 39.16 ± 1.253% and the normal gingiva group (control group #1) with 0.2700 ± 0.5835. Table 3 shows that the test group had the highest significant percentage (Figure 6).

Statistical analysis showed a significant difference among all groups (*p* > 0.0001). The mean number of new blood vessels was significantly higher in the test group.

The results confirmed that the gingival rh-BMP-2 injection resulted in a significant increase in newly formed blood vessels in the gingival tissue. Moreover, a significant difference was noted among all groups, leading to the rejection of the null hypothesis.

## 4. Discussion

An overexpression of VEGF with rh-BMP-2 in bone has been reported in many studies. However, to our knowledge, no previous study has attempted to determine a direct correlation between rh-BMP-2 and gingival vascularization enhancement. In a cornerstone study, Freitas et al. [21] showed that rhBMP-2, associated with absorbable collagen sponge inlays, preserved the alveolar ridge height at extraction socket sites, whereas untreated sites were lost. Jung et al. [22] evaluated the effect of the BMP-7 gene-inducing bone marrow-derived mesenchymal stem cells (BM-MSCs) in periodontal tissue regeneration and confirmed the efficacy of BMP-7 and engineered BMSCs for periodontal tissue regeneration. Statistically significant differences were noted between the efficacy of the 0.75 mg/mL and 1.50 mg/mL rhBMP-2 doses during clinical observations of patients needing extraction socket augmentation. Overall, 25% of implant sites in the 0.75 mg/mL group showed insufficient bone growth at the extraction socket, compared to 56.25% in the 1.50 mg/mL group [23].

Scalzone A et al. [24] showed that the test group (injected with the rh-BMP-2 during the implant placement) displayed a high VEGF marker expression in 80% of the samples, and a significantly higher new vessel formation than both control groups (control group #2: without rh-BMP-2 injection and control group #1: normal gingiva). These results confirm that rh-BMP-2 injection enhanced the new gingival vascularization.

Maxillary alveolar reconstruction in four patients presenting a unilateral cleft lip and palate with or without autologous bone graft with rh-BMP-2 graft showed similar results regarding bone graft volume and height, although the hospitalization length was reduced [24]. In addition, Kalay, E. et al. [25] BMP-2 enhanced angiogenesis by stimulating VEGF activity. Angiogenesis is a crucial mechanism for the initiation of new bone formation and maintenance [25]. In our study, the injection of BMP-2 enhanced the formation of new blood vessels in gingival samples.

The biomechanical conditions for ideal bone formation are not sufficient when angiogenesis stimulation is unfavorable [25]. Pulkkinen, H.H. et al. [26] have shown that BMP-6 modulates VEGF signaling by regulating VEGFR-2 expression and acts via the Hippo signaling effector TAZ. The Hippo signaling regulates cell survival/proliferation, and it is dysregulated in cancer [25]. In a Matrigel plug assay in nude mice, BMP-6 induced angiogenesis [26]. BMP-6 is the first member of the BMP family found to directly regulate both Hippo signaling and new vessel formation [26]. However, to our knowledge, no previous study has attempted to establish a direct correlation between rh-BMP-2 and gingival vascularization enhancement. Our analyses demonstrated that the rh-BMP-2 group had a significantly higher mean new blood vessel formation than the control groups.

According to Zhang et al. [27], associating grafting material with VEGF and BMP-2 might be a potential clinical strategy for tissue engineering, especially in bone reconstruction, due to its ability to deliver growth factors effectively and efficiently.

Liu et al. [28] showed that the slow sequential release of BMP-2 and VEGF promotes osteoblast differentiation and vascular endothelial cell proliferation.

Geng et al. [29] reported that rh-BMP-2 and VEGF-A modRNAs synergistically activate osteogenic and angiogenic programs, resulting in superior healing properties. Also, exploiting chemically modified mRNAs, together with biomaterials, constitutes a potential approach for the clinical treatment of bone injury and defects.

Based on the literature, it seems that the efficient use of bioactive compounds like BMPs may significantly improve bone tissue regeneration. The best kinetic release profile and dosage are still being determined, as well as which combination of molecules is best suited in a particular situation [29].

Alveolar ridge and maxillary sinus augmentation with rh-BMP and collagen sponge seem to be a potential alternative to autogenous bone transplants [29].

Alveolar ridge preservation employing rh-BMP and delivery systems is a suitable pre-treatment for implant therapy in the future [29]. In clinical conditions, the combination between BMP and barrier membrane has the potential to improve periodontal tissue regeneration or even serve as a bone substitute for periodontal regeneration [29].

Several published studies highlighted the effect of BMPs on periodontal healing and inflammation. James et al. [23] demonstrated clinically significant alveolar bone, cementum regeneration, and regulation of periodontal inflammation with the use of rh-BMP-2. Unfortunately, there is a gap in the literature regarding the direct effect of rh-BMP-2 on new blood vessel formation in gingival tissue.

Zhang et al. [30] found that VEGF and BMP-2 act as homing molecules inducing the differentiation of MSCs into endothelial and osteogenic cells. Porous silk scaffolds served as suitable matrix vehicles to release VEGF and BMP-2 in vivo, and bone defects were repaired by promoting both angiogenesis and new bone formation. These findings suggest that combined treatment with VEGF and BMP-2 could be a promising strategy for clinical bone regeneration [30].

Samee, M et al. [31] found he combination of BMP-2 and VEGF significantly increased bone formation, and VEGF transfection resulted in more blood vessels than in the conditions without VEGF. Thus, VEGF might enhance BMP-2-induced bone formation through angiogenesis modulation [31].

The way BMP influences vascular development and function is still not well understood, and existing data indicate that the complexity of BMP signaling extends to the vascular system [32]. Vascular development depends on the regulation of cell junctions linking the endothelial cell system [32].

Several studies of vascular diseases mentioned the links between BMP and proper blood vessel formation and function [32,33]. The results of these studies showed that BMP-2 helps to regulate processes in the body and enhances the formation of small new blood vessels and angiogenesis, promoting the formation of small new blood vessels from existing ones. In addition, BMP-2 is also known to promote cell proliferation and differentiation, cell adhesion, and extracellular matrix formation [33].

Comparing the results from studies on the effects of BMP-2 on the formation of new blood vessels in different organs, such as the heart, brain, or liver, with our results allowed us to explore the different roles of BMP-2 in angiogenesis within distinct biological contexts. BMP-2 might play a role in organ development, regeneration, or injury repair, affecting blood vessel formation accordingly.

In the gingival tissue, BMP-2 may have an effect on wound healing, periodontal regeneration, or dental implant success, influencing blood vessel formation in this specific tissue. These findings may have broader implications in the treatment of organ-specific diseases or injuries where angiogenesis is crucial, like stroke recovery or cardiac regeneration. Studying the effect of BMP-2 on blood vessel formation in different organs and in the gingival tissue highlights the versatility of BMP-2 in regulating angiogenesis within distinct biological contexts. The clinical implications and challenges vary between these studies, emphasizing the importance of tailored research for different medical and dental applications.

A study by Lowery et al. [32] showed that BMP-2 can have significant positive effects on new blood vessel formation, depending on the tissue microenvironment, as it can promote the formation of arterioles, capillaries, and venules [32,33]. David, L. et al. [33] explore the effect of rh-BMP-2 on neovascularization initiation in human gingival tissue holds significant promise for both clinical and research domains. Neovascularization plays a crucial role in various physiological and pathological conditions, particularly in wound healing and tissue regeneration. Rh-BMP-2 influences this process in the human gingival tissue and can have profound implications for dental and periodontal treatments, as well as applications in regenerative medicine for two reasons: First, it may shed light on the mechanisms underlying neovascularization in gingival tissue, providing valuable insights into the complex cellular and molecular pathways involved. Second, it may pave the way for the development of targeted therapies using rh-BMP-2 to enhance the formation of new blood vessels in the gingival region, facilitating the treatment of gum diseases, improving implant success, and accelerating tissue regeneration after surgical procedures.

Future studies are required to investigate the molecular mechanisms underlying the role of BMP-2 in angiogenesis within the gingiva, to determine the optimal dosage of BMP-2 administration to promote new blood vessel formation, and to evaluate the efficacy of combined therapies and the potential of synergistic effects of BMP-2 with other growth factors or angiogenic agents.

The limitation in this study was the lack of research about the best posology of rh-BMP-2 and its effects with the age of the patients. Further research must be conducted regarding these parameters to understand well the efficacity of rh-BMP-2.

## 5. Conclusions

Our results demonstrated that the injection of 1 ml of rh-BMP-2 (0.25 µg/mL) into the gingival flap and grafted material during GBR for dental implantation significantly enhances the formation of neovascularization by the expression of growth factors like VEGF and may increase the healing process of grafted sites.

## Figures and Tables

**Table 1:** Percentage of brown staining intensity and purple staining presence in each sample. Brown: new blood vessel formation. Purple: existing blood vessel.

Color Intensity	Purple Staining	Weak Brown Staining	Moderate Brown Staining	Strong Brown Staining
Normal gingiva (control group #1)	100%			
GBR without rh-BMP-2 (control group #2)		80% (*n* = 16)	20% (*n* = 4)	
GBR with Rh-BMP-2 (test group)			20% (*n* = 4)	80% (*n* = 16)

**Table 2:** Mean total count of blood vessels and standard (Std) deviation of each group. Different superscript letters (A, B, C) indicate a statistically significant difference, while identical superscript letters indicate the absence of a statistically significant difference (*p*-value < 0.0001).

	Normal Gingiva	Without BMP-2	With BMP-2
Number of values	100	100	100
Mean	51.14 ^A^	78.07 ^B^	86.71 ^C^
Std. Deviation	2.391	1.903	1.838

**Table 3:** The mean and standard (Std) deviation of the number of new blood vessels formed in each group are shown. Friedman tests for non-parametric and repetitive measurements coupled to Dunn’s multiple comparisons test (ad hoc test) showed significant differences among all groups. Different superscript letters (A, B, C) indicate a statistically significant difference, while identical superscript letters indicate the absence of a statistically significant difference (*p*-value < 0.0001). BMP-2: bone morphogenic protein 2.

	Normal Gingiva	Without BMP-2	With BMP-2
Number of values	100	100	100
Mean	0.2700 ^A^	39.16 ^B^	49.80 ^C^
Std. Deviation	0.5835	1.253	1.809

## Data Availability

Data are contained within the article.
